# Case report: A novel combination of anomalies in a patient with 22q11.2 deletion syndrome

**DOI:** 10.3389/fped.2023.1298652

**Published:** 2023-11-29

**Authors:** Connor Byeman, Ravi Ashwath

**Affiliations:** Department of Pediatric Cardiology, University of Iowa Stead Family Children’s Hospital, Iowa City, IA, United States

**Keywords:** 22q11.2 deletion, crisscross pulmonary artery, cervical aortic arch, coarctation of the aorta, ventricular septal defect, atrial septal defect, DiGeorge syndrome, conotruncal defects

## Abstract

A frequently occurring genetic disorder, 22q11.2 deletion syndrome can manifest with various abnormalities. The range of cardiac anomalies associated with this syndrome is extensive, with conotruncal defects being the most prevalent. In this study, we report the case of a patient with a unique combination of anatomical abnormalities such as crisscross pulmonary arteries, a cervical aortic arch with coarctation of the aorta, and a ventricular septal defect. The patient underwent initial surgical intervention, which resulted in significant clinical improvement.

## Introduction

22q11.2 deletion syndrome is a prevalent chromosomal microdeletion disorder, occurring in approximately 1 in 3,000–6,000 live births. The genes affected by this syndrome play a crucial role in the development of pharyngeal pouches, and cardiac abnormalities are commonly observed (1). Conotruncal defects, in particular, often aid in the diagnosis (2), especially in early infancy, before overt symptoms of hypocalcemia or immunodeficiency related to thymic aplasia appear.

The deletion displays a diverse range of presentations, including cardiac manifestations. While tetralogy of Fallot and truncus arteriosus represent the most severe forms of associated congenital heart diseases, an interrupted aortic arch or coarctation, pulmonary atresia, crisscross pulmonary arteries, and septal defects are also frequently observed (3). In the case of the patient reported in this study, a unique and complex combination of crisscross pulmonary arteries, left cervical arch with severe coarctation, an aberrant right subclavian artery, and both atrial and ventricular septal defects were present.

## Case report

A female neonate was born at 37 weeks 4 days from uncomplicated vaginal delivery and was admitted promptly to the intensive care unit (ICU) for urgent cardiovascular evaluation. In the prenatal period, the patient was referred for fetal echocardiography, which demonstrated a large ventricular septal defect (VSD) with a concern for coarctation of the aorta. This study did not elucidate any evidence of a hypoplastic thymus. However, APGAR scores were 7 and 8 at 1 and 5 min, respectively. Birthweight was 3.015 kg. Oxygen saturation was noted to be 91% on room air; otherwise, initial vitals were within normal limits. A physical examination demonstrated a 2/6 holosystolic murmur at the lower left sternal border, which was consistent with the prenatal diagnosis of VSD as well as generally syndromic facies. There was no cleft lip or cleft palate. Prostaglandins were started promptly, but there was no need for oxygen supplementation. A microarray was ordered alongside a genetics consultation, given the physical examination findings that showed a documented congenital heart disease. The results in the following days provided a diagnosis of 22q11.2 deletion syndrome.

**Figure 1:**
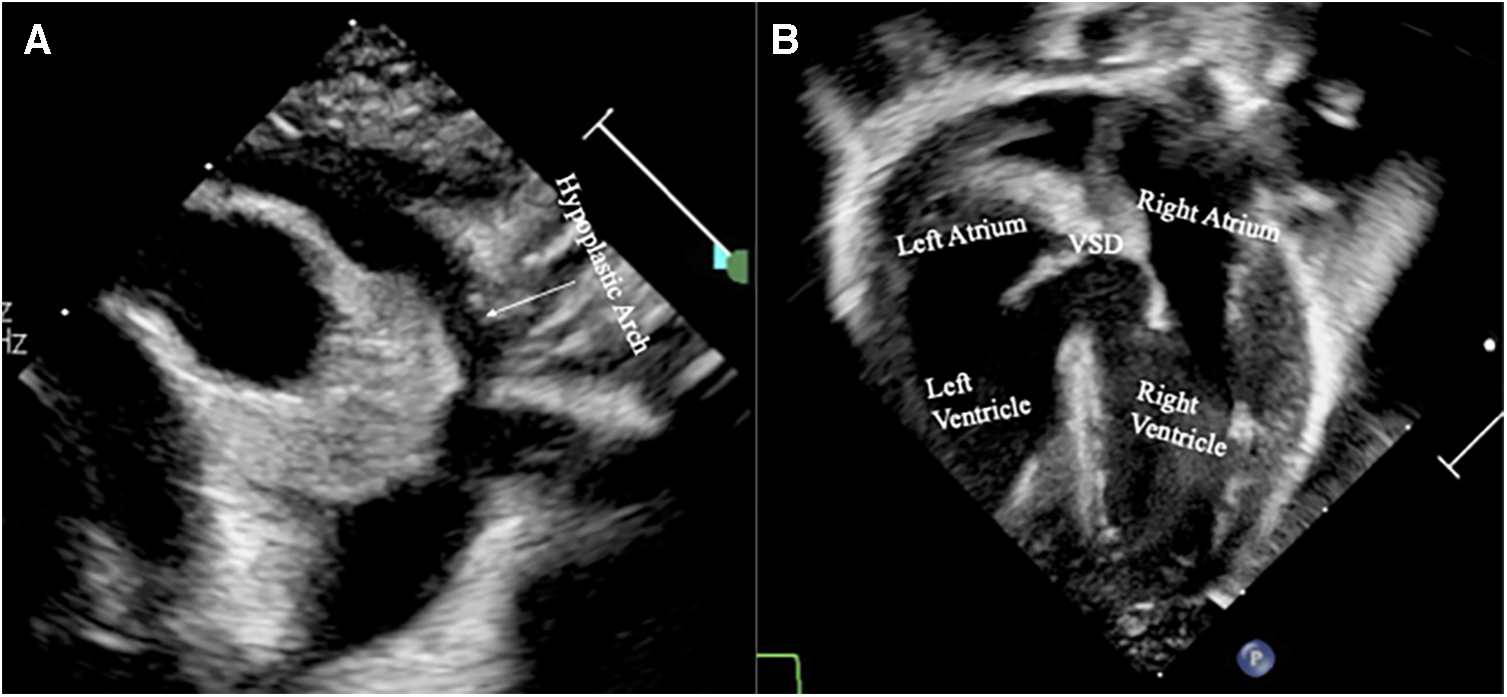
Echocardiographic images obtained shortly after birth demonstrating (**A**) a severe hypoplasia of the ascending aorta and its arch, and (**B**) a substantial muscular ventricular septal defect (VSD).

Transthoracic echocardiography (TTE) was performed on the patient shortly after her admission to the ICU. The results demonstrated a ductal-dependent circulation with a hypoplastic aortic arch with a narrowed distal arch and isthmus, a significantly aneurysmal patent ductus arteriosus (PDA), a muscular VSD with a noticeable bidirectional shunt measuring 0.54 cm, and a dilated main pulmonary artery with crisscross branch pulmonary arteries where the right pulmonary artery arose from the left side and crossed underneath the left pulmonary artery. The left pulmonary artery originated at the expected location. There was an atrial septal defect (ASD) with a minor left to right shunt. The echocardiographic results can be seen in Figure 1.

**Figure 2:**
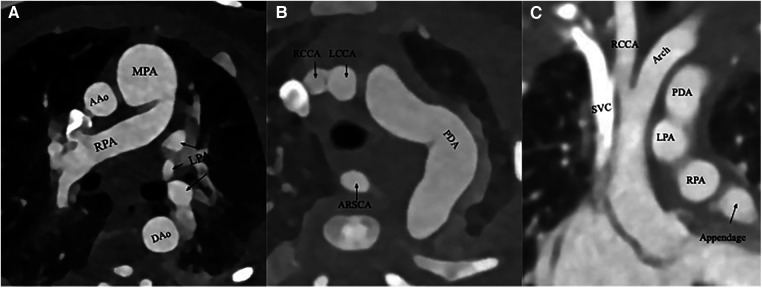
(**A**) An axial CTA slice depicting the right pulmonary artery (RPA) crossing the left pulmonary artery (LPA), which had already branched from the main pulmonary artery (MPA). The ascending (AAo) and descending (DAo) aortic segments are also seen. (**B**) An axial CTA slice demonstrating a substantial and tortuous patent ductus arteriosus (PDA) as well as an aberrant right subclavian artery (ARSCA), originating from the PDA. The other branch vessels such as the right common carotoid arteries (RCCA) and left common carotoid arteries (LCCA) are also seen. (**C**) A coronal CTA slice demonstrating hypoplasia of the ascending aorta and its arch. Several other structures are also seen: the PDA, RCCA, left pulmonary artery (LPA), and right pulmonary artery (RPA), and the superior vena cava (SVC).

To better delineate the anatomy in preparation for surgical repair, a computed tomography angiography (CTA) of the chest was obtained. CTA confirmed the echocardiographic diagnoses, demonstrating severe hypoplasia of the aortic arch with a large and tortuous PDA with crisscross pulmonary arteries. In addition, there was evidence of a previously unknown aberrant right subclavian artery (ARSA) originating from the proximal descending aorta at the junction of the aorta and the PDA. The images of these anatomical abnormalities can be seen in Figure 2.

**Figure 3:**
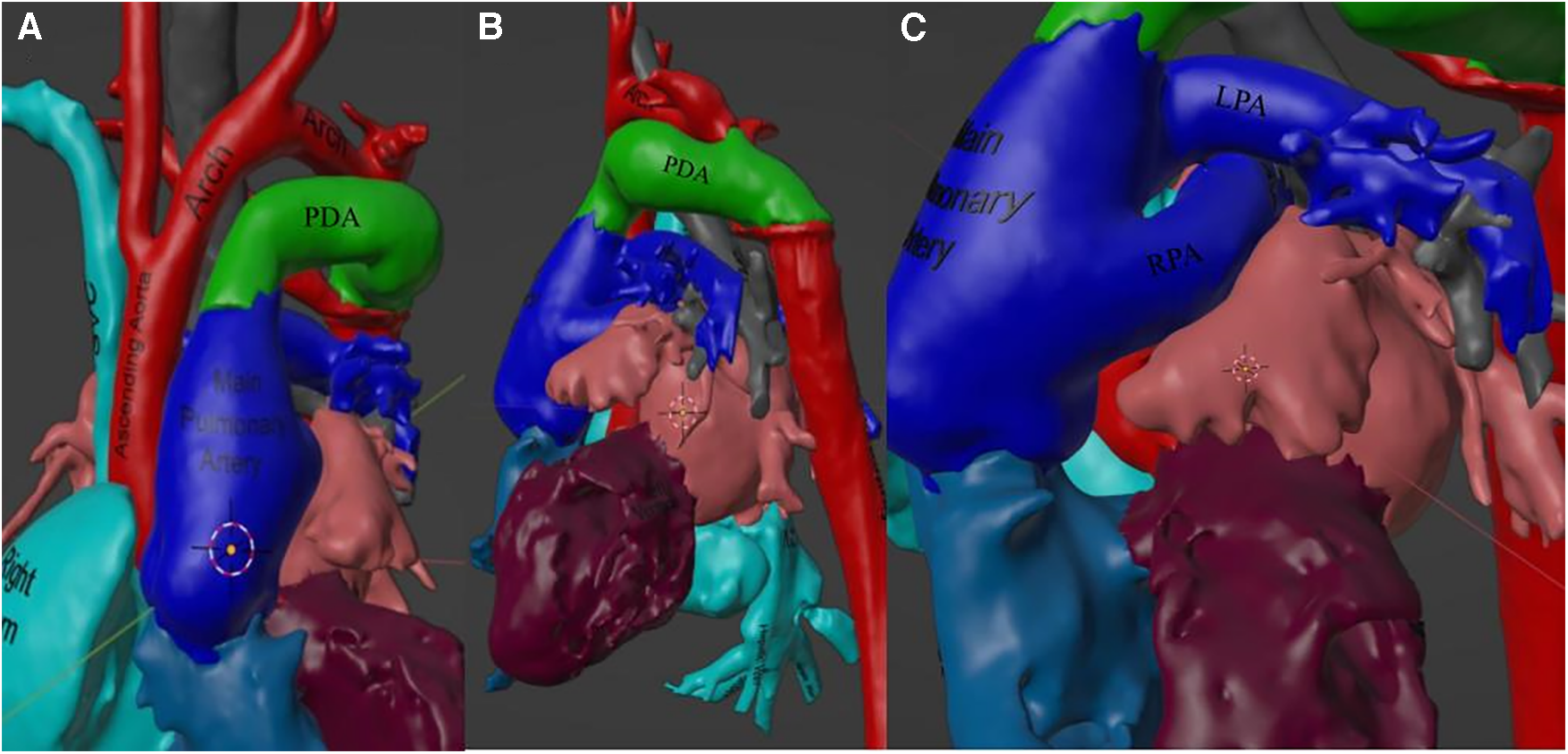
This figure depicts the 3D model used in surgical planning and understanding of the patient's anatomy. (**A**) Visualization of the aortic hypoplasia in 3D space. (**B**) A view from the left of the large patent ductus arteriosus (PDA) feeding the descending aorta. (**C**) A view from the left of the RPA crossing underneath the LPA as well as a scannable augmented reality code to access this 3D model from one's mobile device.

Given the complexity of the patient’s anatomy, it was felt that the heart team would greatly benefit from advanced imaging technology. At this point, a three-dimensional (3D) model was created using Materialise Mimics software and postprocessed using Materialise 3-Matic. The model was used extensively by both the pediatric cardiothoracic surgery and the pediatric cardiology teams in planning. Visuals of the areas of importance in the model are provided in Figure 3.

Operative repair took place on day 6 of life. Sternotomy was performed, and while dissecting through the anterior mediastinum, it was noted that thymic tissue was absent. The pericardium was harvested and placed in glutaraldehyde for later use. A gross inspection revealed a severely hypoplastic ascending arch and pulmonary congestion. The ascending aorta, arch, its vessels, and PDA were completely dissected. The PDA was noted to be incredibly large and tortuous, originating from the main pulmonary artery. A cervical arch pattern was apparent. The patient was placed on cardiopulmonary bypass and the PDA was ligated. The aorta was cross-clamped, and the heart was arrested with cold hyperkalemic antegrade blood cardioplegia. Selective antegrade cerebral perfusion was initiated following the acquisition of vessel control.

The aberrant right subclavian artery was looped and left intact on the descending aorta. The ductus was divided and resected. The isthmus was oversewn, and the proximal descending aorta was reapproximated to the lesser curvature of the transverse arch along the posterior walls. The anterior wall of the anastomosis was patch-augmented with a pulmonary homograft patch. A right atriotomy was performed along the right atrioventricular groove. The muscular VSD was exposed and closed using an autologous pericardial patch. Cross-clamping was removed, and the heart was reperfused with no evidence of arrhythmia. However, a transesophageal echocardiography (TEE) revealed a worsening of the gradients that lie between the ascending aorta and the umbilical line, and an epicardial echocardiography demonstrated a stenosis of the aortic arch just proximal to the junction of the patch augmentation.

A decision was made to restart cardiopulmonary bypass, and as done previously, cardioplegia and antegrade perfusion was initiated following repeat aortic cross-clamping. The proximal aspect of the pulmonary homograft patch was opened at the stenotic junction and additional patch material was placed. Again, perfusion was restored without any evidence of arrhythmia, with TEE demonstrating no remaining gradient. The total cardiopulmonary bypass time was 230 min, cross-clamp time was 117 min, and antegrade cerebral perfusion time was 77 min.

**Figure 4:**
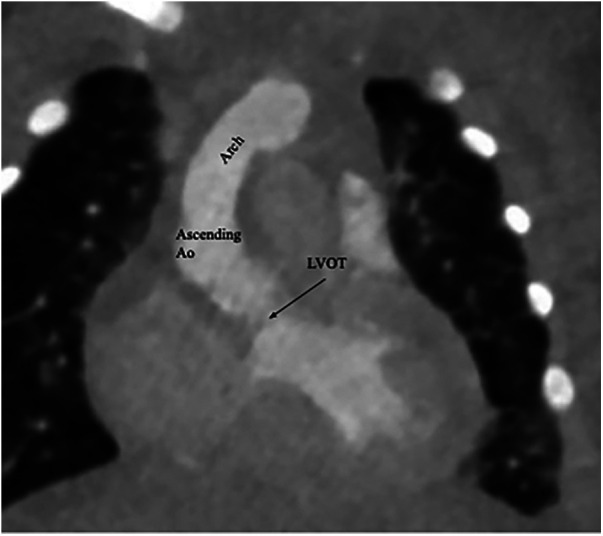
A postrepair coronal CTA demonstrating an intact left ventricular outflow tract (LVOT) as well as an appropriately sized ascending aorta and arch.

Following surgery, the patient required epinephrine and milrinone to maintain a robust biventricular function. Her postoperative period was complicated by moderate pulmonary edema and distributive shock. Over time, it was possible to wean her from pressor support and milrinone. Eventual extubation was successful. Given her protracted clinical course, a repeat CTA was performed, and evidence of coarctation repair can be seen in Figure 4. TTE prior to discharge demonstrated preserved systolic biventricular function with no demonstrable gradients.

## Discussion and conclusion

Congenital heart disease is present in approximately 75%–80% of individuals with 22q11.2 deletion syndrome (4). The wide range of presentations can vary from mild, insignificant VSDs to severe cyanotic lesions such as Tetralogy of Fallot with multiple aortopulmonary collaterals.

While it is not uncommon for individuals with this microdeletion to have one or two commonly associated cardiac anomalies, the case of the patient presented in this study, who had a cervical left aortic arch with an aberrant right subclavian artery, associated severe coarctation, crisscross pulmonary arteries, and a large muscular VSD, is exceedingly rare, and has not been reported previously. Crisscross pulmonary arteries have been reported in 10%–50% of individuals with 22q11.2 deletion syndrome (5, 6). Previous reports (7) have suggested that the identification of crisscross pulmonary arteries should prompt genetic work-up because of its high prevalence in 22q11.2 deletion. In addition, while prenatal ultrasound could not detect the aberrant right subclavian artery in our patient, individuals with non-isolated ARSA detected in this manner are also documented to have a higher risk of chromosomal anomalies, including 22q11.2 deletion (8). Our case provides further evidence that crisscross pulmonary arteries and ARSA should be considered a core abnormality associated with this syndrome. Interestingly, the left pulmonary artery is typically involved in the crisscross arrangement, whereas in our patient, it was the right pulmonary artery that exhibited the aberrant origin.

Given the complexity of the patient's anatomy and the need for precise surgical repair, the utilization of advanced 3D modeling techniques is of utmost importance. Studies have shown that computed tomography (CT) imaging provides a higher level of understanding in cases of 22q11.2 deletion compared with TTE (9). Although traditional 2D modalities such as CT accurately display the anatomy, the creation of 3D models from the original CT scans allows for complete 3D manipulation during surgical planning, providing improved spatial detail.

## Data Availability

The original contributions presented in the study are included in the paper/Supplementary Material; further inquiries can be directed to the corresponding author.

## References

[1] McDonald-McGinn DM. 22q11.2 Deletion syndrome: a tiny piece leading to a big picture. Am J Med Genet A. (2018) 176(10):2055–7. 10.1002/ajmg.a.40653 30380195 PMC6472263

[2] Goldmuntz E Clark BJ Mitchell LE Jawad AF Cuneo BF Reed L Frequency of 22q11 deletions in patients with conotruncal defects. J Am Coll Cardiol. (1998) 32(2):492–8. 10.1016/S0735-1097(98)00259-9 9708481

[3] Momma K. Cardiovascular anomalies associated with chromosome 22q11.2 deletion syndrome. Am J Cardiol. (2010) 105(11):1617–24. 10.1016/j.amjcard.2010.01.333 20494672

[4] Unolt M Versacci P Anaclerio S Lambiase C Calcagni G Trezzi M Congenital heart diseases and cardiovascular abnormalities in 22q11.2 deletion syndrome: from well-established knowledge to new frontiers. Am J Med Genet A. (2018) 176(10):2087–98. 10.1002/ajmg.a.38662 29663641 PMC6497171

[5] Recto MR Parness IA Gelb BD Lopez L Lai WW. Clinical implications and possible association of malposition of the branch pulmonary arteries with DiGeorge syndrome and microdeletion of chromosomal region 22q11. Am J Cardiol. (1997) 80(12):1624–7. 10.1016/S0002-9149(97)00782-0 9416954

[6] Chen J Feng Y. A rare case of crossed pulmonary arteries in an infant—case report. J Cardiothorac Surg. (2013) 8(79):2. 10.1186/1749-8090-8-79 23577830 PMC3639035

[7] Cairello F Gagliardi M Magrassi SA Secco A Strozzi MC Felici E. Crossed pulmonary arteries and DiGeorge syndrome: case reports and literature review. Cardiol Young. (2022):1–2. 10.1017/S1047951122000221 35193728

[8] Cai M Lin N Fan X Chen X Xu S Fu X Fetal aberrant right subclavian artery: associated anomalies, genetic etiology, and postnatal outcomes in a retrospective cohort study. Front Pediatr. (2022) 10:895562. 10.3389/fped.2022.895562 35722491 PMC9203729

[9] Cuturilo G Drakulic D Krstic A Gradinac M Ilisic T Parezanovic V The role of modern imaging techniques in the diagnosis of malposition of the branch pulmonary arteries and possible association with microdeletion 22q11.2. Cardiol Young. (2013) 23(2):181–8. 10.1017/S1047951112000571 22717372

